# Hypoxic Pilates Intervention for Obesity: A Randomized Controlled Trial

**DOI:** 10.3390/ijerph17197186

**Published:** 2020-09-30

**Authors:** Kyounghwa Jung, Jisu Kim, Hun-Young Park, Won-Sang Jung, Kiwon Lim

**Affiliations:** 1 Department of Physical Education, Konkuk University, 120 Neungdong-ro, Gwangjin-gu, Seoul 05029, Korea; pilateslab@konkuk.ac.kr; 2 Physical Activity and Performance Institute (PAPI), Konkuk University, 120 Neungdong-ro, Gwangjin-gu, Seoul 05029, Korea; kimpro@konkuk.ac.kr (J.K.); parkhy1980@konkuk.ac.kr (H.-Y.P.); jws1197@konkuk.ac.kr (W.-S.J.); 3 Department of Sports Medicine and Science, Graduate School, Konkuk University, 120 Neungdong-ro, Gwangjin-gu, Seoul 05029, Korea

**Keywords:** hypoxia, Pilates intervention, vascular endothelial function, hemorheological function, obesity

## Abstract

This study examined the effect of Pilates training under hypoxia, a novel treatment method, for obesity. Thirty-two Korean women with obesity (age: 34–60 (47.5 ± 7.5) years) were randomly assigned to control (CON; *n* = 10), normoxic Pilates training (NPTG; *n* = 10), and hypoxic Pilates training groups (HPTG; *n* = 12). The NPTG and HPTG performed 50 min of Pilates training using a tubing band for 12 weeks (3 days/week) in their respective environmental conditions (NPTG: normoxic condition, inspired oxygen fraction (F_i_O_2_) = 20.9%; HPTG: moderate hypoxic condition, F_i_O_2_ = 14.5%). The CON maintained their daily lifestyle without intervention. All subjects underwent body composition, blood pressure, arterial stiffness, vascular endothelial function, cardiometabolic biomarker, hemorheological function, and aerobic performance measurements before and after the intervention. The HPTG showed a significant improvement in diastolic blood pressure, total cholesterol and triglyceride concentrations, flow-mediated dilation, and erythrocyte deformability and aggregation (all *p* < 0.05) compared with the CON and NPTG. However, compared with the CON and NPTG, the HPTG did not show improvement in other parameters. Hypoxic Pilates intervention is a novel and successful method for promoting endothelial and hemorheological functions in women with obesity.

## 1. Introduction

Obesity, a phenomenon caused by excessive body fat accumulation, is recognized as a global health problem and is reported to increase the prevalence and risk of death because it is a major cause of various metabolic and cardiovascular diseases [1]. According to the 2016 World Health Organization report, 39% of the world’s adult population (>1.9 billion) was overweight and 13% of the population (650 million) was obese [2].

The prevalence rate of obesity in South Korea is increasing rapidly with age (20s: 24.9%, 30s: 33.4%, 40s: 35.3%, 50s: 38%, 60s: 38%, and over 70s: 34.7%) [3].

Physical activity is an effective method for treating obesity because it reduces body fat by reducing subcutaneous and visceral fat [4]. Generally, the most common exercise modality applied for treating obesity is moderate intensity continuous training (MICT), which consists of performing moderate intensity exercise continuously without rest for a long period (≥30 min) using a treadmill [5]. However, MICT has the potential to increase the risk of damage to the musculoskeletal system by overloading knee joints in subjects with obesity who have difficulty in supporting their own excessive weight [6].

Therefore, Pilates is widely used as an alternative exercise modality for obesity treatment [7,8,9]. Pilates, a series of exercise modalities comprising approximately 500 movements inspired by beauty gymnastics, yoga, and ballet, characteristically activates muscles based on the overall perception of the human body by emphasizing control, breathing, flow, accuracy, and concentration [7,10]. Pilates can be performed in a variety of methods and using numerous tools (mat, props, gym ball, foam baller, band, and pillar ring); however, Pilates using a tubing band has the advantage of minimizing the possibility of injury to the joints and muscles and ability to adjust the exercise intensity freely according to ones’ muscle strength and physical fitness [10,11]. However, Pilates is known to have little effect on the metabolic, cardiopulmonary, and vascular functions owing to an exercise load lower than that of MICT [10,12].

Recently, hypoxic exercise, a novel treatment method for obesity, is being widely used to achieve greater metabolic, cardiopulmonary, vascular, and hemorheological functions and lower the mechanical load during physical activity [6,13,14]. Exercise under hypoxia imposes less stress on the locomotor system, while resulting in similar physiological stress [1,6,10]. It induces an increase in the metabolic rate with enhanced carbohydrate oxidation, an additive effect on vascular endothelial function through increased secretion of various vasodilators and vascular endothelial growth factor, leading to the enhancement of metabolic function, cardiac function, arterial compliance, vasodilation, and microcirculation [1,10,13,15]. Hence, sustained exercise under hypoxia might be a promising physical activity modality for successful prevention and treatment of obesity and obesity-related diseases.

However, no study has examined the effects of chronic Pilates intervention under hypoxia on health-related function in a population with obesity. Jung et al. [10] verified that acute Pilates training under moderate hypoxic conditions leads to a greater cardiometabolic response and an additive effect on vascular endothelial function than those under normoxic conditions in subjects with Pilates experience. However, although this study proved the acute effect of Pilates under hypoxia in healthy subjects, it is different from chronic intervention in subjects with obesity.

Therefore, we conducted a randomized controlled trial to investigate the effects of a 12-week Pilates intervention under moderate normobaric hypoxia (inspired oxygen fraction, F_i_O_2_: 14.5%) versus normoxia (F_i_O_2_: 20.9%) on body composition, blood pressure (BP), arterial stiffness, vascular endothelial function, cardiometabolic biomarkers, hemorheological parameters, and aerobic performance in women with obesity.

## 2. Materials and Methods

### 2.1. Subjects

**Figure 1:**
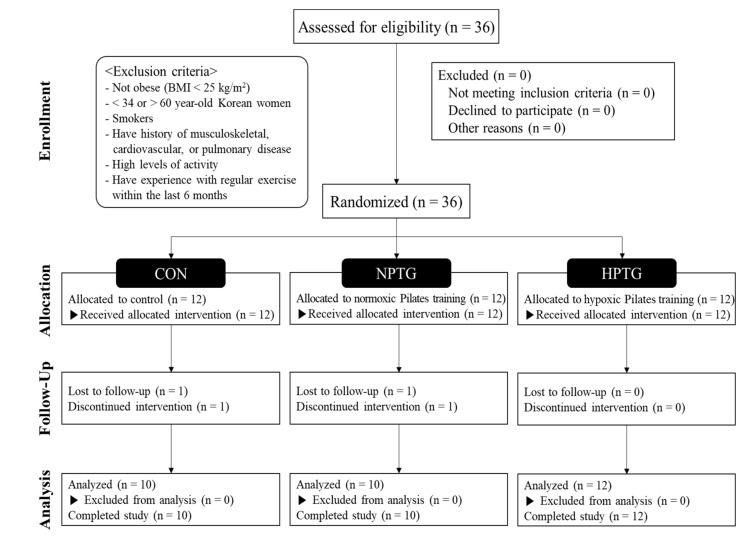
CONSORT (Consolidated Standards of Reporting Trials) flow diagram. CON = control group, HPTG = hypoxic Pilates training group, NPTG = normoxic Pilates training group.

This study included 36 Korean women with obesity (age: 34–60 (47.5 ± 7.5) years) who were non-smokers and had no history of musculoskeletal, diabetes, cardiovascular, or pulmonary disease. All subjects fulfilling the following conditions were included: did not take any medication; had a body mass index (BMI) of >25; participated in only low levels of activity (no exercise performed over the last 6 months). All subjects received information regarding the purpose and process of this study and provided informed consent after receiving sufficient explanation regarding the experiment and possible adverse effects prior to the start of the study. Thereafter, they were randomly assigned into control (CON; *n* = 12), normoxic Pilates training (NPTG; *n* = 12), and hypoxic Pilates training groups (HPTG; *n* = 12) using a computerized random number generator. However, four subjects (CON: 2 and NPTG: 2) were excluded from the study owing to withdrawal (*n* = 2) and noncompliance (*n* = 2). The required sample size was estimated using an effect size (ES = 0.69) reported in the effect of exercise on flow-mediated dilation (FMD) of vascular endothelial function [10]. With an alpha of 0.05 and a desired power of 0.80, the total sample size necessary to achieve statistical significance was estimated to be 12 participants. The sample size calculation was performed using G*Power software (version 3.1.9.7, University of Kiel, Kiel, Germany). The consolidated standards of reporting trial (CONSORT: Consolidated Standards of Reporting Trials) flow diagram is shown in Figure 1 and characteristics of the subjects are presented in Table 1. The trial was reviewed and approved by the Institutional Review Board of the Konkuk University (7001355-201909-HR-334) and was conducted in accordance with the Declaration of Helsinki. The trial information is registered with the Clinical Research Information Service in Korea (KCT0004517).

### 2.2. Study Design

**Figure 2:**
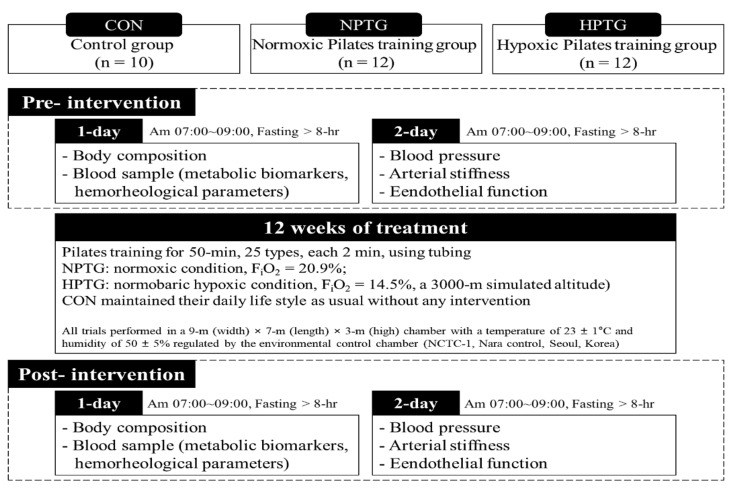
Study design. CON = control group, HPTG = hypoxic Pilates training group, NPTG = normoxic Pilates training group, F_i_O_2_ = inspired oxygen fraction.

The study design was as follows: a 2-day pretesting session (i.e., testing for 2 days with 1-day rest interval), followed by a 12-week intervention and a 2-day post-testing session (Figure 2).

On the first pre- and post-testing days, all subjects fasted for >8 h and after stabilization, a venous blood sample was collected between 7:00 and 9:00 am for the analysis of cardiometabolic biomarkers and hemorheological parameters. Thereafter, their body composition and aerobic performance were measured. On the second pre- and post-testing days, BP, arterial stiffness, and vascular endothelial function were measured in order in the morning after fasting for at least 8 h.

During the intervention period, the NPTG and HPTG subjects performed 50 min of Pilates training using a tubing band in their respective environmental conditions (NPTG: normoxic condition, F_i_O_2_: 20.9%; HPTG: moderate normobaric hypoxic condition, F_i_O_2_: 14.5%, a 3000-m simulated altitude). On the contrary, the CON subjects maintained their daily lifestyle without any intervention. Pilates training using a tubing band comprised 25 types of exercises, each performed in sequence for 2 min. Pilates motion is configured as follows: roll-up and -down, biceps, arm circles, teaser, rolling like a ball, spine twist and arm extension, tubing mermaid, cobra, swimming, double kicks and arm circles, swan, cat, thigh stretching, hug a tree, squat, row, saw, hip pull, the hundred, lats pull three way, leg arc, scissor, helicopter, right side leg pull, and left side leg pull [10].

All subjects underwent body composition (height, weight, free fat mass: FFM, body fat percentage, bone mineral density: BMD, and bone mineral content: BMC), BP (systolic BP: SBP, diastolic BP: DBP, mean arterial pressure: MAP, and pulse pressure: PP), arterial stiffness (brachial-ankle pulse wave velocity: baPWV), vascular endothelial function (pulse wave velocity: PWV), cardiometabolic biomarker concentrations (total cholesterol: TC, high-density lipoprotein cholesterol: HDL-C, low-density lipoprotein cholesterol: LDL-C, triglyceride: TG, free fatty acid: FFA, glucose, insulin, homeostasis model assessment of insulin resistance: HOMA-IR, and homeostasis model assessment of β-cell function: HOMA-β), hemorheological parameters (erythrocyte deformability and erythrocyte aggregation), and aerobic performance (estimated maximal oxygen uptake: VO_2_max) measurements before and after the 12-week intervention.

In all trials, the Pilates program was performed in a 9 × 7 × 3 m (width × length × height) environmental control chamber (NCTC-1, Nara control, Seoul, Korea) at a temperature of 23 ± 1 °C and a humidity of 50 ± 5% [16,17].

### 2.3. Body Composition

The body composition parameters (height, weight, FFM, percent body fat, BMD, and BMC) of all the subjects were analyzed using a dual-energy X-ray absorptiometry scanner (Primus, Osteosys, Seoul, Korea), with their entire body in the center of the examination table, both feet slightly rotated inward, and the shoulders and waist immobile [18].

### 2.4. Blood Pressure

After the subjects sufficiently rested for >20 min, BP in their right brachial artery was measured twice using an autonomic BP monitor (HBP-9020, Omron, Tokyo, Japan) and the average value was used as a result value. If the results of the first and second measurements were different, the measurement was repeated after a 10-min rest.

### 2.5. Arterial Stiffness

After the subjects rested for at least 20 min, resting baPWV in the brachial artery was measured using an automatic oscillometric device (VP-1000plus, Omron, Osaka, Japan). This instrument simultaneously records the baPWV and brachial and ankle BPs on the left and right sides, produces an electrocardiogram, and records the heart sounds. Electrocardiography electrodes were placed on both wrists and cuffs were placed on the brachium and ankles bilaterally. A microphone for detecting heart sounds was placed on the left edge of the sternum. The cuffs were connected to both a plethysmographic sensor, which determined the volume pulse form, and an oscillometric pressure sensor, which measured BP. The brachial and ankle pulse volume waveforms were recorded using a semiconductor pressure sensor. The baPWV values on the right and left sides were obtained and averaged for analysis.

### 2.6. Vascular Endothelial Function

Flow-mediated dilation (FMD) refers to the dilation (widening) of an arteriole caused by the release of nitric oxide (NO) from endothelial cells when blood flow increases in that artery. To evaluate the vascular endothelial function, we measured the FMD of the branched artery using noninvasive Doppler ultrasound (UNEX-EF, Tokyo, Japan) before and after the 12-week intervention. We fixed the ultrasound instrument in the brachial artery region 3–5 cm above the elbow and then measured the diameter of the medial muscle artery. After the measurement, blood was removed for 5 min by increasing BP by 50 mmHg from resting BP. After 5 min, deflation was automatically recorded for next 2 min and the calculated values of FMD (FMD = [reactive hyperemia diameter − baseline diameter] × 100%) were used to evaluate the diameter and blood flow rate were used.

### 2.7. Cardiometabolic Biomarkers

All cardiometabolic biomarkers (TC, HDL-C, LDL-C, TG, FFA, glucose, insulin, HOMA-IR, and HOMA-β) were analyzed by the Seegene medical foundation (an organization certified by the Korea government). The concentrations of TC, HDL-C, LDL-C, TG, FFA, glucose, and insulin were quantified. An 8-mL sample of venous blood was collected into a serum separating tube (SST) for serum. Clot formation was ensured in the SST by centrifuging the sample at 3500 rpm for 10 min. The TC concentration was determined by an enzyme kinetic assay using the Cobas C702 (Roche, Mannheim, Germany). The HDL-C and LDL-C concentrations were detected by homogeneous enzymatic colorimetric assay using the Cobas C702 (Roche, Mannheim, Germany). The glucose concentration was determined by an enzyme kinetic assay using the Cobas8000 C702 (Roche, Mannheim, Germany) and the insulin concentration was detected by an electrochemiluminescence immunoassay using the Cobas8000 e602 (Roche, Mannheim, Germany). HOMA-IR and HOMA-β were calculated using the following formulas: HOMA-IR = (glucose [mg/dL] × insulin [µU/mL])/405 and HOMA-β = (360 × insulin [µU/mL])/(glucose [mg/dL] − 63].

### 2.8. Hemorheological Function

We measured erythrocyte deformability and aggregation as hemorheological function parameters to evaluate microcirculation function. Uyuklu et al. [19] indicated that erythrocyte deformability and aggregation should be analyzed at 25 °C and 3 Pa shear stress within 4–6 h of collecting blood; therefore, all samples were analyzed within 3 h of their collection at room temperature (25 °C) using a Rheoscan-D (Rheo Meditech Inc., Seongbuk-gu, Seoul, Korea) [20]. Erythrocyte deformability was evaluated using the elongation index (EI) by the following process: after transferring the sample to a 2-mL microfuge tube, it was diluted in 700 μL of 5.5% polyvinylpyrrolidone (360 kDa) dissolved in 1 mmol phosphate buffered saline (pH: 7.4; osmolality: 300 mOsmol/kg) in a K3-ethylenediaminetetraacetic acid tube (Greiner bio-one, Chon Nuri, Thailand). Thereafter, 0.5 mL of this solution was analyzed using a D-test kit according to the manufacturer’s instructions (Rheo Meditech Inc.). The accuracy of the RBC EI was measured using a Lineweaver–Burk plot model [21]. Erythrocyte aggregation was evaluated using the aggregation index (AI) by the following process: 8 μL of the whole blood sample was analyzed using an A-test kit according to the manufacturer’s instructions (Rheo Meditech Inc.) [20].

### 2.9. Aerobic Performance

Estimated VO_2_max, considered an aerobic performance parameter, was measured by the graded exercise test using an electrically braked cycle ergometer (Aerobike 75XLIII, Konami, Japan). A heart rate (HR) sensor was attached to the earlobe, and each subject’s information (height, weight, and age) was entered into a bicycle ergometer. Thereafter, following a break until HR stabilization, subjects performed the exercise. All subjects exercised on the bicycle ergometer at a rate of 50 rpm until the HR reached 75% maximal heart rate (HRmax) by the Lamb protocol (15 watt/min; men HRmax: 206 − 0.69 × age, women HRmax: 205 − 0.75 × age). VO_2_max was calculated using the regression equation (VO_2_ = 9.386 watt + 289.6) [22].

### 2.10. Statistical Analysis

Means and standard deviation were calculated for each primary dependent parameter. The normality of distribution of all outcome parameters was verified using the Shapiro–Wilk W-test prior to the parametric tests. A two-way analysis of covariance (“group” × “time”) with repeated measures for time-dependent pretest value was performed to analyze the effects of the 12-week intervention on each dependent parameter. If a significant interaction or main effect within time was found, a Bonferroni post-hoc test was performed to identify within-group change over time. Additionally, the paired *t*-test was performed to compare the post-intervention versus pre-intervention values of dependent parameters in each group separately. We used Cohen’s d (effect size), which reflects the value of a statistic calculated from a sample of data and standardized mean differences. Statistical difference in the means (effect size, Cohen’s d) was determined with significance level (*p* < 0.05) and 95% confidence interval (CI). Cohen′s d effect size (small d = 0.2, medium d = 0.5, and large d = 0.8 effect sizes) were used to assess the significant effects. All analyses were performed using SPSS Statistics 21.0 (IBM Corp., Armonk, NY, USA).

## 3. Results

### 3.1. Pre- and Post-Intervention Data for Body Composition Measures with the Main Analysis of Covariance Results

No significant interaction was noted for any of the body composition parameters, however significant main effects within time were found for weight (*p* = 0.032, *η*^2^ = 0.154) and BMI (*p* = 0.022, *η*^2^ = 0.173) (Table 2). However, post-hoc analysis revealed no significant difference in weight and BMI due to the 12-week intervention.

### 3.2. Pre- and Post-Intervention Data for Blood Pressure and Brachial-Ankle Pulse Wave Velocity Measures with the Main Analysis of Covariance Results

Table 3 depicts the pre- and post-intervention BP and baPWV data of all groups. No significant interaction was noted for any of the BP parameters and baPWV; however, significant main effects within time were noted for SBP (*p* = 0.006, *η*^2^ = 0.244), DBP (*p* = 0.001, *η*^2^ = 0.326), and MAP (*p* = 0.001, *η*^2^ = 0.324). Post-hoc analysis revealed a significant decrease in DBP (Cohen’s d: −0.65, 95% CI: −1.48, 0.24, *p* < 0.05) in the HPTG. However, post-hoc analysis revealed no significant difference in SBP and MAP due to the 12-week intervention.

### 3.3. Pre- and Post-Intervention Data for Cardiometabolic Biomarker Measures with the Main Analysis of Covariance Results

The pre- and post-intervention concentrations of cardiometabolic biomarkers in all groups are presented in Table 4. A significant interaction was found for TG (*p* = 0.005, *η*^2^ = 0.315). Additionally, significant main effects within time were found for TC (*p* < 0.001, *η*^2^ = 0.390), FFA (*p* < 0.001, *η*^2^ = 0.429), and glucose (*p* = 0.020, *η*^2^ = 0.177). Post-hoc analyses revealed a significant decrease in the TC (Cohen’s d: −0.44, 95% CI: −1.27, 0.43, *p* < 0.05) and TG (Cohen’s d: −0.48, 95% CI: −1.31, 0.39, *p* < 0.05) concentrations in the HPTG and a significant increase in the glucose concentration in the NPTG (Cohen’s d: 0.91, 95% CI: −0.01, 1.75, *p* < 0.05).

### 3.4. Pre- and Post-Intervention Measures of the Endothelial Function Parameters with the Main Analysis of Covariance Results

**Figure 3:**
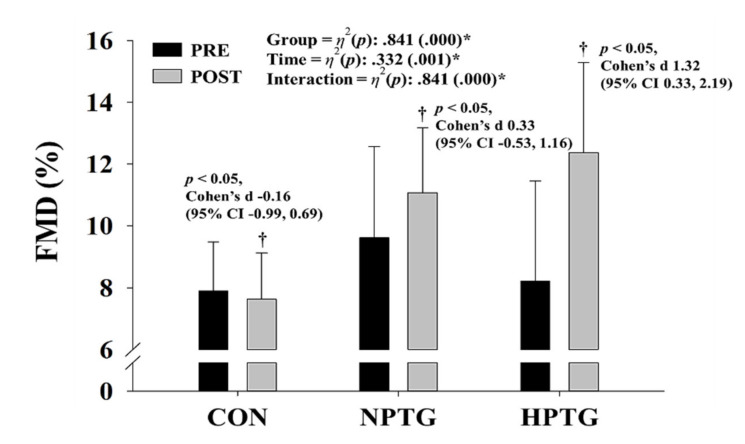
Pre- and post-intervention measures of the endothelial function parameters with the main analysis of covariance results. CI = confidence interval, CON = control group, FMD = flow-mediated dilation, HPTG = hypoxic Pilates training group, NPTG = normoxic Pilates training group. * *p* < 0.05 significant interaction or main effect within time. † *p* < 0.05 significant difference between pre- and post-intervention in each group.

A significant interaction was found for FMD (*p* < 0.001, *η*^2^ = 0.841), which corresponds to vascular endothelial cell function (Figure 3). Post-hoc analyses revealed significant attenuation of FMD in the CON (Cohen’s d: −0.16, 95% CI: −0.99, 0.69, *p* < 0.05) and significant enhancement of FMD in the NPTG (Cohen’s d: 0.33, 95% CI: −0.53, 1.16, *p* < 0.05) and HPTG (Cohen’s d: 1.32, 95% CI: 0.33, 2.19, *p* < 0.05). However, a greater improvement in FMD was observed in the HPTG than in the NPTG (NPTG vs. HPTG: 15.6% vs. 51.2%).

### 3.5. Pre- and Post-Intervention Measures of the Hemorheological Function Parameters with the Main Analysis of Covariance Results

**Figure 4:**
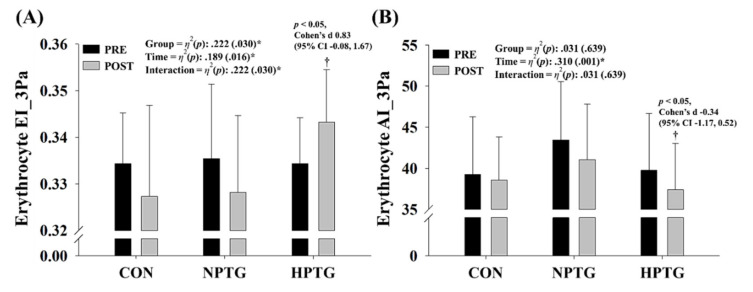
Pre- and post-intervention measures of the hemorheological function parameters with the main analysis of covariance results. (**A**) Erythrocyte deformability (**B**) Erythrocyte aggregation, AI = aggregation index, CI = confidence interval, CON = control group, EI = elongation index, HPTG = hypoxic Pilates training group, NPTG = normoxic Pilates training group. * *p* < 0.05 significant interaction or main effect within time. † *p* < 0.05 significant difference between pre- and post-intervention in each group.

Figure 4 depicts the pre- and post-intervention erythrocyte EI and AI data corresponding to hemorheological function for all groups. A significant interaction was found for the erythrocyte EI_3 Pa (*p* < 0.030, *η*^2^ = 0.222). Additionally, a significant main effect within time was found for the AI_3 Pa (*p* < 0.001, *η*^2^ = 0.310). Post-hoc analyses revealed a significant improvement in the erythrocyte EI_3 Pa (Cohen’s d: 0.83, 95% CI: −0.08, 1.67, *p* < 0.05) and erythrocyte AI_3 Pa (Cohen’s d: −0.34, 95% CI: −1.17, 0.52, *p* < 0.05) in the HPTG.

### 3.6. Pre- and Post-Intervention Data for Aerobic Performance Measures with the Main Analysis of Covariance Results

Aerobic performance of all groups is presented in Table 5. No significant interaction and main effect within time were found for estimated VO_2_max. Compared with the control and normoxic Pilates intervention, the moderate hypoxic Pilates intervention for 12 weeks had no effect on aerobic performance (estimated VO_2_max).

## 4. Discussion

In this study, compared with the 12-week Pilates intervention under normoxia, the 12-week Pilates intervention under moderate hypoxia (F_i_O_2_: 14.5%) enhanced the endothelial and hemorheological functions in women with obesity. Additionally, hypoxic Pilates training improved DBP and the TC and TG concentrations. However, no changes were found in body composition and aerobic performance.

Hypoxic training has been designed to improve the performance of individual- and team-sports athletes [23,24,25,26]. Recently, hypoxic training is being widely used for preventing and treating obesity and obesity-related clinical problems such as hypertension, diabetes, metabolic diseases, and cardiovascular diseases [1,5,6,13]. Alternatively, hypoxic training enhances the aerobic performance and reduces the obesity and obesity-related diseases by improving aerobic metabolic process (e.g., enhanced oxygen transportation and utilization capacity) [5,13,25,26,27]. Generally, exercise under moderate hypoxia is an effective method for weight loss and improving body composition through various physiological responses and adaptations such as an increase in activity of appetite-related gut hormones and adipocytokines, a change in energy substrate utilization, an increase in the energy metabolic rate, a reduction in perception of hunger feeling and in food intake, and an increase in the activity of various endocrine factors [1,27,28]. Additionally, hypoxic training increases the secretion of insulin, insulin-like growth factor-1, erythropoietin, and sex hormones such as androgens and testosterone, which increase the muscle mass and basal metabolic rate [5,29,30,31]. Based on these rationales, previous studies have verified the effectiveness of a 4–12-week aerobic exercise under moderate hypoxia (F_i_O_2_: 16.5–14.5%, 2000–3000 m simulated altitude) on body composition [1,15,32]. However, no weight loss and improvement in body composition due to the 12-week Pilates intervention under moderate hypoxia was noted in the present study. This difference in findings is thought to be influenced by two factors. First, we did not investigate the dietary intake and daily activity in each group during the 12-week intervention period. Therefore, we could not consider the effects of dietary intake and daily activity on changes in body weight and composition. Second, we considered that the difference of the exercise intensity and type between the Pilates program performed in the present study and the exercise program performed in previous studies had an effect. The majority of previous studies reporting weight loss and improvement in body composition involved performance of moderate-intensity aerobic exercise under moderate hypoxia using a treadmill or bicycle. However, our study applied a low-intensity Pilates training program under moderate hypoxia. It has been reported that low-intensity and low-impact exercise types do not affect body composition and bone metabolism [18]. Hence, we consider that the difference in the exercise program and intensity is the main reason for the difference in results.

Exercise under hypoxia results in a higher cardiometabolic response than that under normoxia for similar workloads [6,33]. The absolute proportion of carbohydrate oxidation and its relative contribution to the fuel mixture at similar workloads are enhanced under hypoxia compared to those under normoxia. Hypoxic exercise elicits an additional cardiometabolic stress that the body must overcome to maintain the energy supply within activating muscles [10,33]. Additionally, hypoxia induces a variety of cellular and metabolic responses such as hypoxic inducible factor-1 and vascular endothelial growth factor-1 expression, angiogenesis, increase in glycolytic enzymes and number of mitochondria, improvement in insulin sensitivity, and increase in glucose transporter-4 [27,34]. Hence, hypoxic exercise has a positive effect on improving cardiometabolic risk factors [35]. Haufe et al. [36] divided 20 healthy men into two groups that performed a similar exercise program (60 min of exercise, three times/week for 4 weeks) at 15% F_i_O_2_ and 21% F_i_O_2_, with the heart rate measured at 3 mmol/L lactate during pre-exercise testing. Consequently, the hypoxic training group showed greater improvements in the TG and insulin concentrations, HOMA-index, and area under the curve for insulin during the oral glucose tolerance test, although the absolute workload under hypoxia (1.4 watts/kg) was lower than that under normoxia (1.7 watts/kg). Wisner et al. [32] reported that exercise (60 min, 3 days/week for 4 weeks) with 65% VO_2_max under moderate hypoxia (F_i_O_2_: 15%) was effective in decreasing body fat mass, insulin level, and HOMA-index, although the absolute exercise load under hypoxia was lower than that under normoxia. In the present study, we confirmed that hypoxic Pilates training was more effective in reducing the TC and TG concentrations than normoxic Pilates training. Our findings on cardiometabolic risk factors are consistent with those of previous studies. However, no outstanding change in other cardiometabolic biomarkers (e.g., HDL-C, LDL-C, FFA, glucose, insulin, HOMA-IR, and HOMA-β) was noted. This is thought to be because of our Pilates program, which did not provide sufficient stimulation to cardiometabolic function, and therefore, had a lower exercise intensity than other aforementioned aerobic exercises.

An exercise training program induces augmented blood flow and subsequently increases vasodilation and upregulates endothelial NO synthase. The release of various vasodilators such as adenosine, prostaglandin, and NO during exercise contributes to the mechanism responsible for vasodilation. Particularly, hypoxia induces an increase in NO bioavailability resulting in vasodilation and a reduction in total systemic vascular resistance and thereby BP [37,38]. Moreover, an exercise under moderate hypoxia induces a reduction in arterial stiffness and the activity of the sympathetic nervous system, relaxation of blood vessels, and improvement of blood flow and hemorheological functions such as erythrocyte deformability and aggregation [10,13,14,38]. In particular, the improvement of erythrocyte deformability and aggregation is very important because it is related to the surrounding micro-circulation tissue and facilitates the exchange of oxygen and carbon dioxide [20]. Nishiwaki et al. [39] investigated the effect of exercise training in 2000-m simulated altitude on arterial stiffness and FMD in postmenopausal women and concluded that exercise under moderate hypoxia may induce vascular functional adaptations such as an increase in FMD response. Muangritdech et al. [38] examined the effects of intermittent hypoxic breathing (F_i_O_2_: 14%) during exercise on BP, NO metabolites, and hypoxia inducible factor-1 alpha (HIF-1α) over a 6-week period. They confirmed that exercise under hypoxia reduces SBP and increases the NO metabolites and HIF-1α concentrations. These results suggest that exercise under moderate hypoxia can be used as an alternative therapeutic strategy for preventing and treating obesity by improving endothelial and hemorheological functions. Based on this mechanism, our present research team previously investigated the effect of an acute Pilates program under moderate hypoxia on vascular endothelial function in Pilates participants [10]. Although the Pilates program was not effective in improving cardiovascular function due to low exercise intensity [12], hypoxic Pilates training elicited an additive effect on vascular endothelial functions such as FMD. Our present study verified the effectiveness of a long-term Pilates intervention under moderate hypoxia based on our previous findings [10]. In the present study, we confirmed that compared with normoxic Pilates training, hypoxic Pilates training for 12 weeks improves BP (DBP), vascular endothelial function (FMD), and hemorheological functions such as erythrocyte deformability and aggregation. Overall, our findings on BP, endothelial function, and hemorheological function after the hypoxic Pilates training are consistent with those of previous studies.

However, although vascular endothelial and hemorheological function improved, no remarkable positive effect of the Pilates intervention under moderate hypoxia on aerobic performance (estimated VO_2_max) was noted. This is because the Pilates intervention performed in the present study had a low positive impact on cardiopulmonary, vascular, and hemorheological functions owing to lower workload than those (moderate-intensity aerobic exercise programs) in previous studies. Therefore, we believe that providing a higher-workload Pilates program for the Pilates intervention under moderate hypoxia will have a more pronounced positive effect on body composition, cardiovascular risk factors, and aerobic performance.

## 5. Limitation

Some limitations of the present study should be considered when interpreting results. The normality of distribution of all outcome parameters was verified using the Shapiro–Wilk W-test prior to the parametric tests, however the small sample size was a limitation to elicit the effectiveness of hypoxic Pilates intervention on improving endothelial and hemorheological function in obese women. In addition, the trial period in the present study was relatively short of 12-weeks, and the dietary intake and daily activity of participants during the intervention period were not investigated [40].

## 6. Conclusions

The 12-week Pilates intervention under moderate hypoxia elicited a decrease in BP and the TC and TG concentrations and an increase in FMD and erythrocyte deformability and erythrocyte aggregation in women with obesity compared with the Pilates intervention under normoxia. However, it did not affect the body composition, arterial stiffness, and aerobic performance owing to a lower workload than that in the moderate intensity aerobic exercise program. Thus, our findings suggest that the 12-week Pilates intervention under moderate hypoxia improved cardiometabolic parameters, vascular endothelial function, and hemorheological function in the present study. The hypoxic Pilates intervention may be an efficient exercise strategy for preventing and treating obesity by improving endothelial and hemorheological functions and minimizing the possibility of injury to joints and muscles.

## Figures and Tables

**Table 1:** Characteristics of subjects.

Variables	Groups			*F*-Value	*η*^2^(*p*) Value
	CON (*n* = 10)	NPTG (*n* = 10)	HPTG (*n* = 12)		
Age (years)	51.6 ± 6.5	43.8 ± 8.6	47.2 ± 6.4	2.962	0.170 (0.068)
Height (cm)	155.7 ± 4.8 ^a^	162.1 ± 5.6 ^b^	158.5 ± 5.4 ^ab^	3.625	0.200 (0.039) *
Weight (kg)	60.5 ± 6.8	65.8 ± 11.0	67.9 ± 11.0	1.513	0.094 (0.237)
BMI (kg/m^2^)	25.2 ± 2.0	25.1 ± 3.3	27.1 ± 4.3	1.266	0.080 (0.297)
FFM (kg)	38.9 ± 4.5	43.2 ± 5.5	41.3 ± 3.9	0.791	0.052 (0.463)
Percent body fat (%)	37.0 ± 4.4	37.8 ± 5.0	40.2 ± 6.4	1.051	0.068 (0.363)

Note. Values are expressed as means ± standard deviation. BMI = body mass index, CON = control group, FFM = free fat mass, HPTG = hypoxic Pilates training group, NPTG = normoxic Pilates training group. * *p* < 0.05 Significant interaction or main effect within time. ^a,b^ Different letters indicate significant difference.

**Table 2:** Pre- and post-intervention data for body composition measures with the main analysis of covariance results.

Variables	Groups	Pre-Test	Post-Test	Cohen′s d (95% CI)	*η*^2^ (*p*) Value	
Weight (kg)	CON	61.1 ± 6.8	61.0 ± 7.1	−0.01 (−0.85, 0.83)	Group	0.031 (0.643)
	NPTG	66.3 ± 11.0	65.8 ± 11.5	−0.04 (−0.87, 0.81)	Time	0.154 (0.032) *
	HPTG	68.0 ± 10.1	68.0 ± 11.4	−0.00 (−0.84, 0.84)	Interaction	0.031 (0.643)
BMI (kg/m^2^)	CON	25.2 ± 2.0	25.1 ± 2.0	−0.03 (−0.87, 0.81)	Group	0.007 (0.902)
	NPTG	25.1 ± 3.3	25.0 ± 3.5	−0.04 (−0.88, 0.80)	Time	0.173 (0.022) *
	HPTG	27.1 ± 4.3	27.1 ± 4.9	−0.00 (−0.84, 0.84)	Interaction	0.007 (0.902)
FFM (kg)	CON	38.9 ± 4.5	38.9 ± 4.9	0.08 (−0.76, 0.92)	Group	0.035 (0.606)
	NPTG	43.2 ± 5.5	43.5 ± 5.5	0.03 (−0.81, 0.87)	Time	0.001 (862)
	HPTG	41.3 ± 3.9	40.8 ± 4.0	−0.01 (−0.85, 0.83)	Interaction	0.035 (0.606)
Percent Body Fat (%)	CON	37.0 ± 4.4	37.4 ± 5.1	0.06 (−0.79, 0.89)	Group	0.055 (0.451)
	NPTG	37.8 ± 5.0	38.0 ± 5.7	0.05 (−0.79, 0.89)	Time	0.093 (0.102)
	HPTG	40.2 ± 6.4	40.1 ± 6.9	−0.03 (−0.87, 0.81)	Interaction	0.055 (0.451)
BMD (g/cm2)	CON	1.12 ± 0.12	1.13 ± 0.12	−0.02 (−0.85, 0.83)	Group	0.062 (406)
	NPTG	1.09 ± 0.10	1.10 ± 0.09	0.05 (−0.79, 0.89)	Time	0.006 (686)
	HPTG	1.12 ± 0.11	1.19 ± 0.11	0.04 (−0.80, 0.88)	Interaction	0.062 (406)
BMC (g)	CON	1986.4 ± 269.8	1982.3 ± 274.4	−0.10 (−0.94, 0.74)	Group	0.023 (0.724)
	NPTG	2117.5 ± 240.2	2132.8 ± 259.9	0.02 (−0.82, 0.86)	Time	0.042 (0.278)
	HPTG	2091.1 ± 226.0	2100.0 ± 237.1	0.12 (−0.73, 0.95)	Interaction	0.023 (0.724)

Note. Values are expressed as means ± standard deviation. BMC = bone mineral content, BMD = bone mineral density, BMI = body mass index, CI = confidence interval, CON = control group, FFM = free fat mass, HPTG = hypoxic Pilates training group, NPTG = normoxic Pilates training group. * *p* < 0.05 Significant interaction or main effect within time.

**Table 3:** Pre- and post-intervention data for blood pressure and brachial-ankle pulse wave velocity measures with the main analysis of covariance results.

Variables	Groups	Before	After	Cohen′s d (95% CI)		*η*^2^ (*p*) Value	
SBP (mmHg)	CON	118.8 ± 16.8	120.0 ± 15.4	0.07 (−0.77, 0.91)		Group	0.089 (0.271)
	NPTG	119.1 ± 9.8	120.4 ± 11.1	0.12 (−0.73, 0.95)		Time	0.244 (0.006) *
	HPTG	122.0 ± 13.1	116.3 ± 10.5	−0.48 (−1.31, 0.39)		Interaction	0.089 (0.271)
DBP (mmHg)	CON	69.9 ± 8.0	68.1 ± 8.5	−0.22 (−1.05, 0.63)		Group	0.021 (0.739)
	NPTG	72.2 ± 10.3	71.2 ± 9.5	−0.10 (−0.94, 0.74)		Time	0.326 (0.001) *
	HPTG	77.3 ± 9.5	71.8 ± 6.3	−0.65 (−1.48, 0.24)	†	Interaction	0.021 (0.739)
MAP (mmHg)	CON	86.2 ± 10.4	85.4 ± 9.9	−0.08 (−0.92, 0.76)		Group	0.040 (0.562)
	NPTG	87.8 ± 9.5	87.6 ± 9.2	−0.03 (−0.87, 0.81)		Time	0.324 (0.001) *
	HPTG	92.2 ± 10.5	86.6 ± 7.2	−0.60 (−1.43, 0.28)		Interaction	0.040 (0.562)
PP (mmHg)	CON	48.9 ± 11.5	51.9 ± 11.5	0.26 (−0.60, 1.09)		Group	0.075 (0.337)
	NPTG	46.9 ± 7.5	49.2 ± 8.9	0.26 (−0.59, 1.10)		Time	0.084 (0.121)
	HPTG	44.8 ± 5.8	44.5 ± 7.0	−0.04 (−0.88, 0.80)		Interaction	0.075 (0.337)
baPWV (cm/s)	CON	1246.7 ± 177.4	1252.2 ± 240.5	0.02 (−0.82, 0.86)		Group	0.058 (0.435)
	NPTG	1189.8 ± 141.5	1183.6 ± 98.7	−0.04 (−0.88, 0.80)		Time	0.045 (0.258)
	HPTG	1261.6 ± 148.5	1202.1 ± 153.8	−0.39 (−1.22, 0.47)		Interaction	0.058 (0.435)

Note. Values are expressed as means ± standard deviations. baPWV = brachial-ankle pulse wave velocity, CI = confidence interval, CON = control group, DBP = diastolic blood pressure, HPTG = hypoxic Pilates training group, MAP = mean arterial pressure, NPTG = normoxic Pilates training group, PP = pulse pressure, SBP = systolic blood pressure. * *p* < 0.05 Significant interaction or main effect within time. † *p* < 0.05 significant difference between pre- and post-intervention in each group.

**Table 4:** Pre- and post-intervention data for cardiometabolic biomarker measures with the main analysis of covariance results.

Variables	Groups	Before	After	Cohen′s d (95% CI)		*η*^2^ (*p*) Value	
Total cholesterol (mg/dL)	CON	190.7 ± 25.7	193.2 ± 22.4	0.10 (−0.74, 0.94)		Group	0.094 (0.253)
	NPTG	191.0 ± 26.7	187.2 ± 20.4	−0.15 (−0.99, 0.70)		Time	0.390 (0.000) *
	HPTG	228.4 ± 58.6	203.4 ± 45.2	−0.44 (−1.27, 0.43)	†	Interaction	0.094 (0.253)
Triglyceride (mg/dL)	CON	108.6 ± 26.8	127.6 ± 28.6	0.68 (−0.21, 1.52)		Group	0.315 (0.005) *
	NPTG	98.4 ± 28.6	83.0 ± 18.0	−0.62 (−1.45, 0.27)		Time	0.196 (0.014) *
	HPTG	135.2 ± 41.7	114.8 ± 43.5	−0.48 (−1.31, 0.39)	†	Interaction	0.315 (0.005) *
HDL-C (mg/dL)	CON	55.9 ± 11.4	55.9 ± 11.5	0.00 (−0.84, 0.84)		Group	0.003 (0.953)
	NPTG	61.3 ± 7.5	60.6 ± 11.0	−0.08 (−0.92, 0.76)		Time	0.020 (0.459)
	HPTG	57.5 ± 14.6	56.4 ± 15.3	−0.07 (−0.91, 0.77)		Interaction	0.003 (0.953)
LDL-C (mg/dL)	CON	117.8 ± 22.6	114.5 ± 22.0	−0.15 (−0.98, 0.70)		Group	0.025 (0.702)
	NPTG	116.9 ± 27.2	104.9 ± 17.7	−0.34 (−1.17, 0.52)		Time	0.025 (0.401)
	HPTG	151.7 ± 52.4	142.0 ± 61.9	−0.16 (−1.00, 0.68)		Interaction	0.025 (0.702)
Free fatty acid (µEq/L)	CON	926.7 ± 290.8	752.5 ± 343.7	−0.54 (−1.38, 0.33)		Group	0.075 (0.337)
	NPTG	854.9 ± 383.6	585.6 ± 191.8	−0.85 (−1.70, 0.06)		Time	0.429 (0.000) *
	HPTG	976.7 ± 525.6	826.1 ± 421.4	−0.31 (−1.14, 0.55)		Interaction	0.075 (0.337)
Glucose (mg/dL)	CON	99.0 ± 19.5	96.9 ± 18.3	−0.11 (−0.95, 0.73)		Group	0.049 (0.494)
	NPTG	86.8 ± 8.5	94.6 ± 8.6	0.91 (−0.01, 1.75)	†	Time	0.177 (0.020) *
	HPTG	103.2 ± 17.9	101.8 ± 17.3	−0.08 (−0.92, 0.76)		Interaction	0.049 (0.494)
Insulin (mg/dL)	CON	6.4 ± 3.3	7.5 ± 4.5	0.25 (−0.60, 1.08)		Group	0.093 (0.256)
	NPTG	5.4 ± 2.0	6.1 ± 2.6	0.28 (−0.58, 1.11)		Time	0.013 (0.549)
	HPTG	8.0 ± 3.1	7.4 ± 2.9	−0.20 (−1.03, 0.65)		Interaction	0.093 (0.256)
HOMA-IR	CON	1.6 ± 1.0	1.9 ± 1.4	0.19 (−0.66, 1.03)		Group	0.101 (0.225)
	NPTG	1.2 ± 0.5	1.4 ± 0.7	0.40 (−0.47, 1.23)		Time	0.006 (0.691)
	HPTG	2.1 ± 1.0	1.9 ± 0.9	−0.19 (−1.02, 0.66)		Interaction	0.101 (0.225)
HOMA-β (%)	CON	70.4 ± 29.4	81.0 ± 25.2	0.38 (−0.48, 1.21)		Group	0.117 (0.176)
	NPTG	84.3 ± 20.2	71.1 ± 28.3	−0.52 (−1.35, 0.36)		Time	0.123 (0.057)
	HPTG	78.4 ± 28.1	75.6 ± 30.9	−0.10 (−0.93, 0.75)		Interaction	0.117 (0.176)

Note. Values are expressed as means ± standard deviations. CI = confidence interval, CON = control group, HDL-C = high density lipoprotein cholesterol, HOMA-β = homeostasis model assessment of β-cell function, HOMA-IR = homeostatic model assessment for insulin resistance, HPTG = hypoxic Pilates training group, LDL-C = low-density lipoprotein cholesterol, NPTG = normoxic Pilates training group. * *p* < 0.05 Significant interaction or main effect within time. † *p* < 0.05 significant difference between pre- and post-intervention in each group.

**Table 5:** Pre- and post-intervention data for aerobic performance measures with the main analysis of covariance results.

Variable	Groups	Before	After	Cohen′s d (95% CI)	*η*^2^ (*p*) Value	
Estimated VO_2_max (mL/min/kg)	CON	23.9 ± 2.6	24.6 ± 4.5	0.17 (−0.68, 1.00)	Group	0.065 (0.392)
	NPTG	25.2 ± 2.9	25.2 ± 2.6	0.01 (−0.83, 0.85)	Time	0.002 (0.814)
	HPTG	24.4 ± 5.3	27.1 ± 8.6	0.34 (−0.52, 1.18)	Interaction	0.065 (0.392)

Note. Values are expressed as means ± standard deviations. CI = confidence interval, CON = control group, HPTG = hypoxic Pilates training group, NPTG = normoxic Pilates training group, VO_2_max = maximal oxygen consumption.
